# Predictors for imaging progression on chest CT from coronavirus disease 2019 (COVID-19) patients

**DOI:** 10.18632/aging.102999

**Published:** 2020-04-10

**Authors:** Zongguo Yang, Jia Shi, Zhang He, Ying Lü, Qingnian Xu, Chen Ye, Shishi Chen, Bozong Tang, Keshan Yin, Yunfei Lu, Xiaorong Chen

**Affiliations:** 1 Department of Integrative Medicine, Shanghai Public Health Clinical Center, Fudan University, Shanghai 201508, China; 2 Department of Neurology, Shanghai Public Health Clinical Center, Fudan University, Shanghai 201508, China

**Keywords:** coronavirus disease 2019, COVID-19, monocyte-lymphocyte ratio, MLR, age

## Abstract

Objective: This study aimed to investigate the potential parameters associated with imaging progression on chest CT from coronavirus disease 19 (COVID-19) patients.

Results: The average age of 273 COVID-19 patients enrolled with imaging progression were older than those without imaging progression (p = 0.006). The white blood cells, platelets, neutrophils and acid glycoprotein were all decreased in imaging progression patients (all p < 0.05), and monocytes were increased (p = 0.025). The parameters including homocysteine, urea, creatinine and serum cystatin C were significantly higher in imaging progression patients (all p < 0.05), while eGFR decreased (p < 0.001). Monocyte-lymphocyte ratio (MLR) was significantly higher in imaging progression patients compared to that in imaging progression-free ones (p < 0.001). Logistic models revealed that age, MLR, homocysteine and period from onset to admission were factors for predicting imaging progression on chest CT at first week from COVID-19 patients (all p < 0.05).

Conclusion: Age, MLR, homocysteine and period from onset to admission could predict imaging progression on chest CT from COVID-19 patients.

Methods: The primary outcome was imaging progression on chest CT. Baseline parameters were collected at the first day of admission. Imaging manifestations on chest CT were followed-up at (6±1) days.

## INTRODUCTION

Since the end of 2019, a novel coronavirus with person-to-person transmission has spread to many other countries worldwide [1–5]. Previous epidemiology report uncovered that the epidemic of coronavirus disease 2019 (COVID-19) has doubled every 7.4 day in its early stage, with an average serial interval of 7.5 days [3]. Early information estimated that the basic reproductive number R_0_ was estimated to be 1.4 – 2.5 reported by WHO [2]. The pandemic is accelerating at an exponential rate and at risk of escalating into a global health emergency [2]. The mortality of coronavirus disease 2019 (COVID-19) patients in China is approximately 2.3%, compared with 9.6% of severe acute respiratory syndrome (SARS) and 34.4% of middle east respiratory syndrome (MERS) reported by WHO [6]. Even this virus is not as fetal as people thought, the transmissibility is far exceeding that of SARS and MERS [7]. Although many clinical and epidemiological literatures have been published [3–6, 8–10], the spread in still ongoing and the early warning parameters for disease progression remain incomplete.

Compared to symptoms, chest CT findings were more rapid and frequent [11, 12]. The imaging performance on chest CT scans from COVID-19 patients mainly manifested as bilateral ground-glass opacities (GGOs) in the lung periphery [13]. In a retrospective cohort, chest CTs of 121 symptomatic COVID-19 patients have been reviewed. Bilateral lung involvement was observed in 10/36 early patients (28%), 25/33 intermediate patients (76%), and 22/25 late patients (88%) [11]. Currently, chest CT is used to assess the severity of lung involvement in COVID-19 pneumonia [14]. In a cohort study, 85.7% (54/63) confirmed COVID-19 patients developed imaging progression including enlarged and increased extent of GGOs and consolidation at early follow-up chest CT scans [12]. That is, short-term imaging progression on chest CT from COVID-19 patients should be early predicted and intervened.

In this analysis, we summarized the baseline characteristics and investigated the potential predictive parameters for imaging progression on chest CT scans at first week after admission of COVID-19 patients, in the hope that the data may provide novel biomarker candidates as well as useful insights into the pathogenesis and progression of COVID-19 patients.

## RESULTS

### Imaging performance of progression and progression-free patients

**Figure 1:**
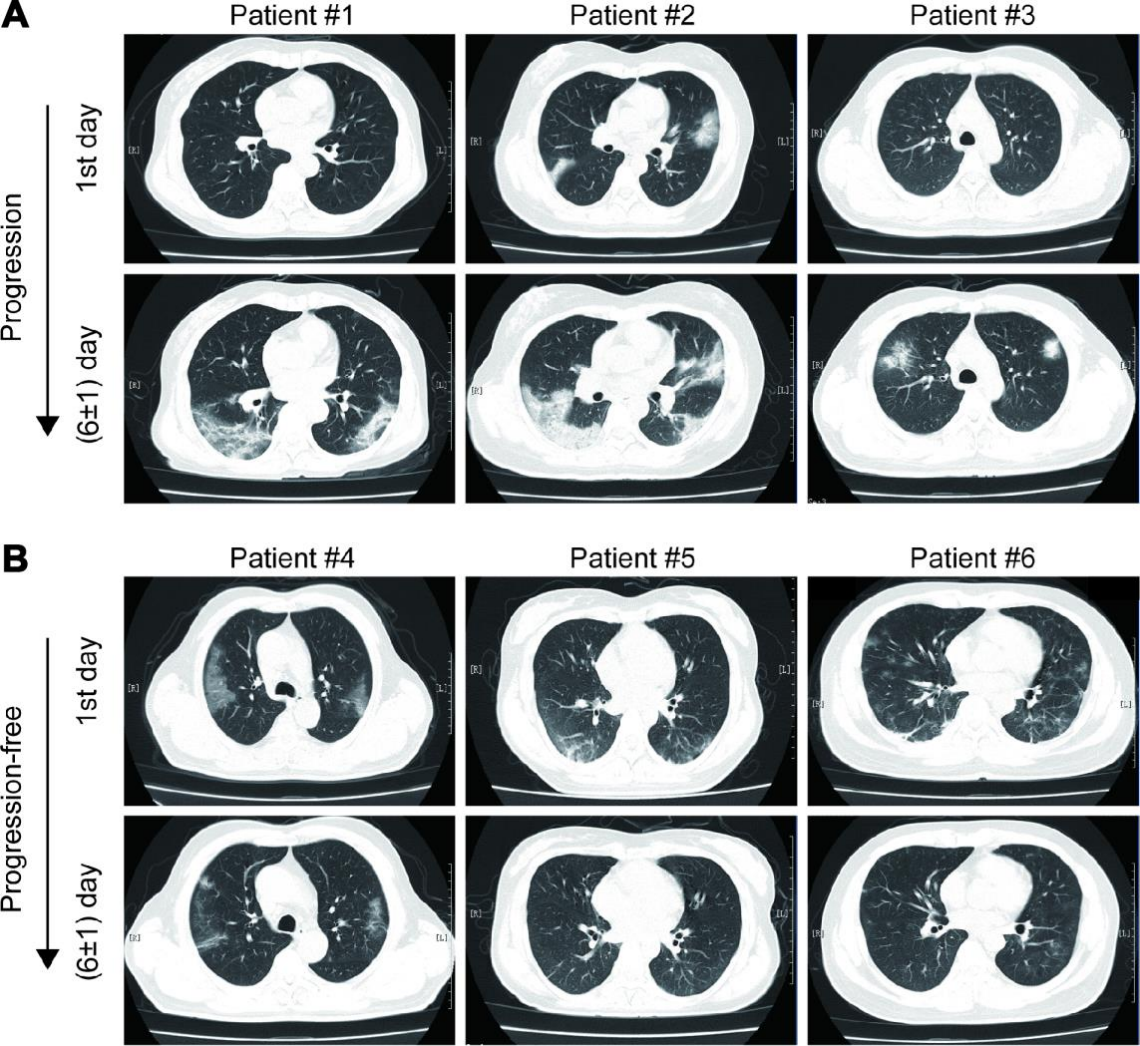
Examples of imaging progression (**A**) and progression-free (**B**) in chest CT from COVID-19 patients.

As shown in Figure 1, most mild type COVID-19 patients had bilateral and peripheral GGOs, consolidation and linear opacities imaging involvements on chest CT at the first admission day. Some patients had no remarkable hallmarks. At the first six (±1) day, enlarged and increased GGOs, consolidation, solid nodules and fibrous stripes were observed for patients suffered from imaging progression on chest CT scans. On the contrary, the GGOs, consolidation and linear opacities were partly resolved and decreased for imaging progression-free patients.

### Baseline characteristics and inflammatory model comparisons between imaging progression and progression-free patients

In total, 71 COVID-19 patients suffered from imaging progression on chest CT at first week after admission, and the other 202 patients were imaging progression-free on chest CT. As summarized in Table 1, the patients in imaging progression group were significantly older than those in imaging progression-free group (p = 0.006, Table 1). More patients were treated with gamma globulin and thymosin in imaging progression group compared to those without imaging progression (p = 0.022 and p = 0.001, respectively, Table 1). In blood routine tests, the white blood cells (WBC), platelets and neutrophils were significantly lower in imaging progression patients than those in imaging progression-free ones (p = 0.025, p = 0.044 and p = 0.014, respectively, Table 1), while the monocytes were significantly higher in imaging progression patients (p = 0.025, Table 1). Additionally, acid glycoprotein was significantly lower in imaging progression patients (p = 0.037, Table 1). In liver function tests, gamma-glutamyl transferase (GGT) levels were significantly higher in imaging progression-free patients (p = 0.045, Table 1), while homocysteine levels were significantly higher in imaging progression patients (p = 0.006, Table1). In kidney function tests, urea, creatinine and serum cystatin C levels were significantly higher in imaging progression patients compared to those in imaging progression-free ones (p = 0.011, p = 0.007, respectively, Table 1). As we expected, the estimated glomerular filtration rate (eGFR) levels were significantly decreased in imaging progression patients (p < 0.001, Table 1). No differences were found in cardiac markers and coagulation function tests.

**Table 1:** Baseline characteristics of COVID-19 patients.

**Variables**	**Chest CT**		**p value**
	**Progression group (n = 71)**	**Progression-free group (n = 202)**	
Age, years, mean ± SD	53.5 ± 1.9	47.6 ± 1.1	0.006
Male, n (%)	33 (46.5)	101 (50)	0.61
Disease history, n (%)			0.614
None	48 (67.6)	143 (70.8)	
Hypertension	13 (18.3)	27 (13.4)	
Diabetes	7 (9.9)	11 (5.4)	
Fatty liver disease	12 (16.9)	27 (13.4)	
Others	3 (4.2)	21 (10.4)	
Epidemiology, n (%)			
Hubei sojourning history	43 (56.3)	108 (53.5)	0.301
Contact with COVID-19 patients	27 (38.0)	72 (35.6)	0.719
Therapeutic strategy, n (%)			
Antivirus drugs	58 (81.7)	141 (69.8)	0.053
Antibiotics	22 (31.0)	46 (22.8)	0.169
Gamma globulin	13 (18.3)	17 (8.4)	0.022
Thymosin	20 (28.2)	23 (11.4)	0.001
Glucocorticoid	10 (14.1)	17 (8.4)	0.169
TCM decoction	5 (7.0)	25 (12.4)	0.216
TCM patent	27 (38.0)	58 (28.7)	0.145
Chest CT imaging, n (%)			0.504
Bilateral lung lesion	60 (84.5)	177 (87.6)	
Single lung lesion	11 (15.5)	25 (12.4)	
Blood routine tests, mean ± SD			
WBC, 10^3^/mm^3^	4.6 ± 0.1	5.2 ± 0.1	0.025
RBC, 10^4^/mm^3^	4.4 ± 0.1	4.5 ± 0.04	0.334
Hemoglobin, g/L	135.1 ± 1.7	136.7 ± 1.1	0.465
Platelet, 10^3^/mm^3^	176.0 ± 6.6	195.0 ± 5.1	0.044
Neutrophils, 10^3^/mm^3^	2.9 ± 0.1	3.5 ± 0.1	0.014
Lymphocytes, 10^3^/mm^3^	1.2 ± 0.1	1.3 ± 0.04	0.342
Monocytes, 10^3^/mm^3^	0.5 ± 0.03	0.4 ± 0.01	0.025
Hypersensitive CRP, mg/L, mean ± SD	17.5 ± 2.4	18.7 ± 1.6	0.697
ESR, mm/Hour, mean ± SD	56.9 ± 4.3	64.5 ± 2.7	0.148
Procalcitonin, ng/ml, mean±SD	0.05 ± 0.01	0.09 ± 0.05	0.687
Acid glycoprotein, mg/dl, mean ± SD	140.9 ± 5.6	154.5 ± 3.3	0.037
Liver function tests, mean ± SD			
ALT, U/L	27.6 ± 2.3	27.6 ± 1.4	0.995
AST, U/L	29.4 ± 1.7	29.2 ± 1.6	0.958
GGT, U/L	29.5 ± 2.5	38.6 ± 2.5	0.045
LDH, U/L	244.4 ± 10.4	248.8 ± 5.8	0.703
TBiL, μmol/L	8.4 ± 0.4	9.2 ± 0.3	0.116
Albumin, g/L	40.8 ± 0.4	41.1 ± 0.3	0.537
Globulin, g/L	28.8 ± 0.5	29.0 ± 0.3	0.693
Homocysteine, μmol/L	10.7 ± 0.5	9.3 ± 0.2	0.006
Renal function test, mean ± SD			
Urea, mmol/L	5.1 ± 0.2	4.5 ± 0.1	0.011
Creatinine, μmol/L	70.7 ± 3.0	63.0 ± 1.3	0.007
Serum cystatin C, mg/L	1.0 ± 0.04	0.8 ± 0.01	< 0.001
eGFR, ml/(min×1.73m^2^)	101.3 ± 3.1	116.3 ± 1.9	< 0.001
Lactic acid, mmol/L, mean ± SD	2.8 ± 0.1	2.8 ± 0.04	0.936
Haptoglobin, mg/dl, mean ± SD	209.2 ± 12.0	229.6 ± 7.0	0.142
Retinol-binding protein, mg/L, mean ± SD	27.8 ± 1.4	26.4 ± 0.7	0.327
Cardiac markers, mean ± SD			
cTnI, ng/ml	0.029 ± 0.004	0.033 ± 0.003	0.455
Myoglobin, ng/ml	17.5 ± 3.0	14.7 ± 2.9	0.59
Pro-BNP, pg/ml	73.5 ± 13.7	67.6 ± 7.2	0.692
Coagulation function tests, mean ± SD			
INR	1.01 ± 0.008	1.02 ± 0.008	0.424
PTA	99.9 ± 1.2	99.0 ± 0.8	0.579
Prothrombin time, second	13.4 ± 0.08	13.5 ± 0.08	0.402
D-Dimer, μg/ml	0.55 ± 0.06	0.77 ± 0.11	0.254

TCM, Traditional Chinese Medicine; WBC, white blood cells; RBC, red blood cells; CRP, C-reactive protein; ESR, erythrocyte sedimentation rate; ALT, alanine aminotransferase; AST, aspartate aminotransferase; GGT, gamma-glutamyl transferase; LDH, lactate dehydrogenase; TBiL, total bilirubin; eGFR, estimated glomerular filtration rate; cTnI, cardiac troponin; Pro-BNP, Brain natriuretic peptide prohormone; INR, international normalized ratio; PTA, prothrombin activity.

**Figure 2:**
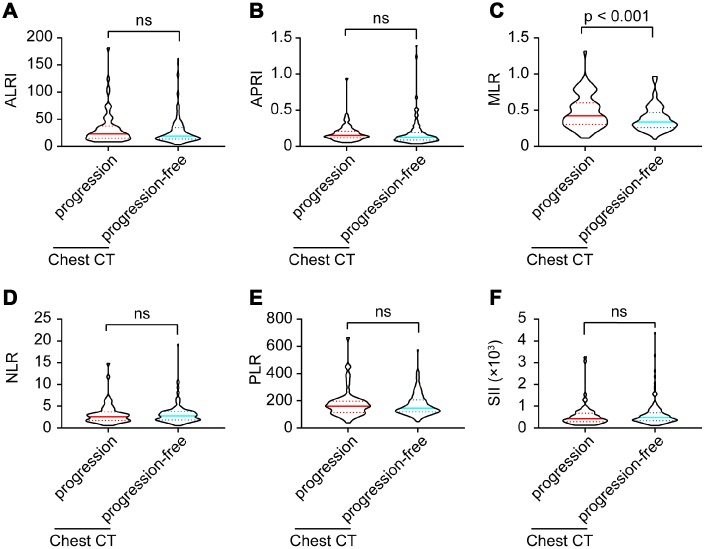
ALRI (**A**), APRI (**B**), MLR (**C**), NLR (**D**), PLR (**E**) and SII (**F**) model comparisons between imaging progression and progression-free COVID-19 patients.

Six inflammatory models were compared between imaging progression and progression-free patients. As shown in Figure 2, monocyte-lymphocyte ratio (MLR) levels were significantly higher in imaging progression patients than those in imaging progression-free ones (p < 0.001, Figure 2C), while no differences were found among aspartate aminotransferase-lymphocyte ratio index (ALRI), aspartate aminotransferase-platelet ratio index (APRI), neutrophil-lymphocyte ratio (NLR), platelet-lymphocyte ratio (PLR) and systemic immune-inflammation index (SII) between these two groups (Figure 2A, 2B, 2D–2F).

### Co-manifestations on chest CT and outcomes

As summarized in Table 2, except for common manifestations on chest CT, chronic inflammatory manifestation, chronic bronchitis / emphysema, pericardial effusion, pleural effusion, bullae of lung and obsolete tuberculosis were the most frequent imaging co-manifestations in COVID-19 patients. COVID-19 patients with imaging progression had significantly higher frequency of chronic inflammatory manifestation than those without imaging progression (12.7% vs. 3.5%, p = 0.005, Table 2). No differences were found in distributions of chronic bronchitis / emphysema, pericardial effusion, pleural effusion, bullae of lung and obsolete tuberculosis between these two groups (Table 2).

**Table 2:** Co-manifestations on chest CT in COVID-19 patients.

**Co-manifestations, n (%)**	**Chest CT**		**p value**
	**Progression group (n = 71)**	**Progression-free group (n = 202)**	
Chronic inflammatory manifestations	9 (12.7)	7 (3.5)	0.005
Chronic bronchitis / emphysema	2 (2.8)	2 (1.0)	0.271
Pericardial effusion	1 (1.4)	1 (0.5)	0.438
Pleural effusion	1 (1.4)	0 (0)	0.091
Bullae of lung	1 (1.4)	2 (1.0)	0.771
Obsolete tuberculosis	2 (2.8)	1 (0.5)	0.107

Moreover, no acute bacterial or other viral co-infection performances on chest CT were found in these COVID-19 patients.

All these COVID-19 patients did not develop severe conditions, no one died during our follow up.

### Parameters associated with imaging progression on chest CT

Variables including age, gender, disease history, epidemiology, chest CT imaging, therapeutic strategies, period from onset to admission, ALRI, APRI, MLR, NLR, PLR, SII, WBC, neutrophils, lymphocytes, monocytes, platelet, red blood cells (RBC), hemoglobin, C-reactive protein (CRP), erythrocyte sedimentation rate (ESR), procalcitonin, alanine aminotransferase (ALT), aspartate aminotransferase (AST), GGT, lactate dehydrogenase (LDH), total bilirubin (TBiL), albumin, globulin, urea, creatinine, eGFR, lactic acid, haptoglobin, acid glycoprotein, cystatin C, homocysteine, retinol-binding protein, cardiac troponin (cTnI), myoglobin, brain natriuretic peptide prohormone (pro-BNP), prothrombin time, prothrombin activity (PTA), international normalized ratio (INR), D-dimer were included in the univariate analysis. As presented in Table 3, age, gamma globulin therapy, thymosin therapy, MLR, serum cystatin C, homocysteine, eGFR and period from onset to admission were potential parameters associated with imaging progression (all p < 0.05, Table 3). When these parameters were included in the multivariate model, age, MLR and homocysteine were significantly correlated with imaging progression on chest CT from COVID-19 patients (RR = 2.28, 95%CI = 1.12 – 4.34, p = 0.012; RR = 7.69, 95%CI = 1.67 – 35.55, p = 0.009 and RR = 3.17, 95%CI = 1.01 – 9.96, p = 0.048; respectively, Table 3). In addition, COVID-19 patients with period from onset to admission ≥ 4 days might have lower risk to develop imaging progression on chest CT at first week after admission (RR = 0.35, 95%CI = 0.19 – 0.67, p = 0.001, Table 3).

**Table 3:** parameters associated with imaging progression in chest CT from COVID-19 patients^#^.

**Variables**	**Univariate**		**p value**		**Multivariate**		**p value**
	**RR**	**95%CI**			**RR**	**95%CI**	
Age, years							
<60	reference	-	1.0		reference	-	1.0
≥60	2.72	1.55-4.78	< 0.001		2.28	1.12-4.34	0.012
Gamma globulin, yes vs. no	2.44	1.12-5.32	0.025		1.08	0.38-3.08	0.89
Thymosin, yes vs. no	3.05	1.55-6.0	0.001		2.32	0.94-5.73	0.069
MLR, per increase 1 unit	12.2	3.09-48.23	< 0.001		7.69	1.67-35.55	0.009
Serum cystatin C, mg/L							
< 1.03	reference	-	1.0		reference	-	1.0
> 1.03	2.8	1.35-5.82	0.006		0.79	0.28-2.2	0.65
Homocysteine, μmol/L							
< 15.4	reference	-	1.0		reference	-	1.0
> 15.4	3.54	1.23-10.14	0.019		3.17	1.01-9.96	0.048
eGFR, ml/(min×1.73m^2^)							
> 90	reference	-	1.0		reference	-	1.0
< 90	2.97	1.54-5.75	0.001		1.63	0.67-4.0	0.281
Period from onset to admission, days							
< 4	reference	-	1.0		reference	-	1.0
≥ 4	0.36	0.20-0.64	0.001		0.35	0.19-0.67	0.001

Variables including age, gender, disease history, epidemiology, chronic inflammatory co-manifestation on chest CT, therapeutic strategies, period from onset to admission, ALRI, APRI, MLR, NLR, PLR, SII, WBC, neutrophils, lymphocytes, monocytes, platelet, RBC, hemoglobin, CRP, ESR, procalcitonin, ALT, AST, GGT, LDH, TBiL, albumin, globulin, urea, creatinine, eGFR, lactic acid, haptoglobin, acid glycoprotein, cystatin C, homocysteine, retinol-binding protein, cTnI, myoglobin, pro-BNP, prothrombin time, PTA, INR, D-dimer were included in the univariate analysis. Only variables with p < 0.05 in univariate model were included in the multivariate analysis. ^#^ Only variables significantly associated with imaging progression in chest CT in univariate analysis were presented.

### Predictive values of MLR and age for imaging progression on chest CT

**Figure 3:**
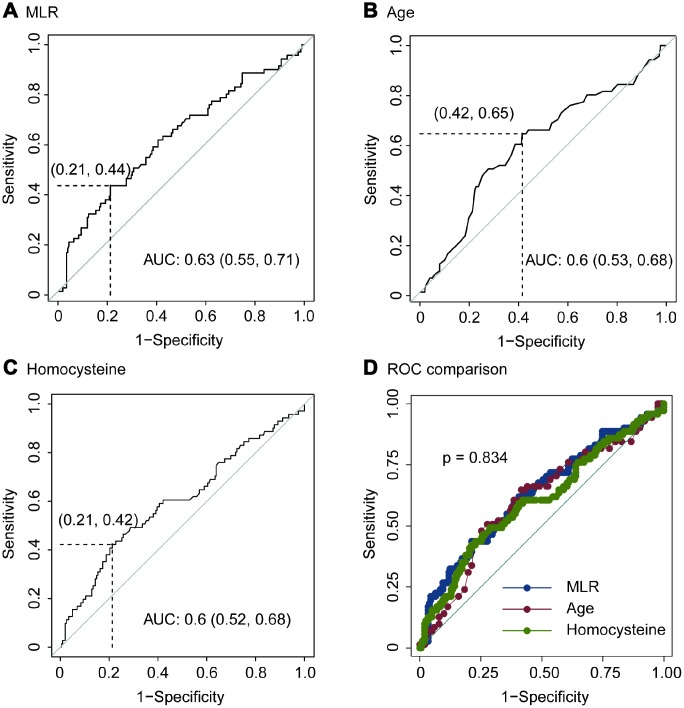
ROC of MLR (**A**), age (**B**), homocysteine (**C**) and ROC comparison (**D**) for imaging progression in chest CT from COVID-19 patients.

Using OptimalCutpoints package in R program, we detected that the optimal cutoff of MLR was 0.51. The sensitivity, specificity, positive predictive value (PPV) and negative predictive value (NPV) of MLR for predicting imaging progression on chest CT were 0.44, 0.79, 0.42 and 0.80, respectively (Figure 3A and Table 4). And, the AUC of MLR for predicting imaging progression on chest CT was 0.63 (Figure 3A).

**Table 4:** Predictive values of MLR model, age and homocysteine for imaging progression on chest CT from COVID-19 patients.

	**Estimate**	**95%CI**
MLR		
Cutoff	0.51	-
Sensitivity	0.44	0.32 – 0.56
Specificity	0.79	0.72 – 0.84
Positive predictive value	0.42	0.34 – 0.54
Negative predictive value	0.80	0.71 – 0.85
Age, years		
Cutoff	51	-
Sensitivity	0.65	0.53 – 0.76
Specificity	0.58	0.51 – 0.65
Positive predictive value	0.35	0.29 – 0.48
Negative predictive value	0.83	0.74 – 0.86
Homocysteine, μmol/L		
Cut off	10.58	
Sensitivity	0.42	0.31 – 0.55
Specificity	0.79	0.72 – 0.84
Positive predictive value	0.41	0.33 – 0.53
Negative predictive value	0.80	0.70 – 0.85

The optimal cutoff of age for predicting imaging progression on chest CT was 51 years. The sensitivity, specificity, PPV and NPV were 0.65, 0.58, 0.35 and 0.83 respectively (Figure 3B and Table 4). ROC curve revealed that the AUC of age in the prediction model was 0.6 (Figure 3B).

In addition, the optimal cutoff of homocysteine for predicting imaging progression on chest CT from COVID-19 patients was 10.58 μmol/L. The sensitivity, specificity, PPV and NPV were 0.42, 0.79, 0.41 and 0.80, respectively (Figure 3C and Table 4).

We performed ROC comparison in MLR, age and homocysteine using ROC regression. As showed in Figure 3D, no difference among these three indexes was found (p = 0.834, Figure 3D).

## DISCUSSION

According to the Chinese guidelines, imaging progression-free on chest CT scans was one of discharge criteria for COVID-19 patients. At present stage, the long-term imaging features of COVID-19 are not yet known [13, 15]. Follow-up imaging in COVID-19 patients often demonstrated the disease progression. Generally, imaging manifestations are in line with the severity of COVID-19 [16]. Hence, a short-term follow up with identification of imaging progression is of great importance for early warning of disease aggravation from COVID-19 patients, which could help clinicians to manage quickly and accurately [12]. Considered that, we defined the imaging progression at first week on chest CT as the primary outcome.

In this outbreak, age was considered as one critical content during the disease occurrence and development. Our results also revealed that the average age of patients with imaging progression was older than those without. Logistic model confirmed that age should be a risk factor for predicting imaging progression. Previous reports suggested that COVID-19 is more susceptible to infect older adults [3, 8, 10]. Research with small samples of 2019-nCoV infected infants have been reported [17]. In a study included 34 COVID-19 children, the authors concluded that the clinical manifestations in children with 2019-nCoV infection are non-specific and are milder than that in adults [18]. In a nationwide retrospective study, 2143 pediatric patients were included. They found that more than 90% patients were asymptomatic, mild, or moderate, even though young children, particularly infants, were vulnerable to infection [19]. The first deaths of COVID-19 occurred frequently among elderly people, who may progress more faster [20]. In a multicenter cohort study with 137 patients enrolled, age was shown to be associated with high risk of death in COVID-19 patients. Middle-aged and elderly patients with underlying comorbidities are prone to respiratory failure and have a poorer prognosis [21, 22]. Combined the previous literatures and our results, we assumed that age also should be a risk factor for imaging progression at the early stage of COVID-19.

Among the six inflammatory models, MLR was significantly higher in COVID-19 patients with imaging progression on chest CT scans, and correlated with imaging aggravation. Previous evidence demonstrated that monocytes/macrophages were susceptible to human coronavirus (HCoV) 229E infection, but strongly restricted OC43 replication [23]. Differs from HCoV-229E, SARS-CoV poorly infects human purified monocytes/macrophages, and production of interferon-alpha by these cells further limits the infection [24]. Following infection of monocytes/macrophages by HCoV-OC43, viability remained high over 6 days and no apoptosis was observed [25]. These clues suggested that monocytes might be stable in function and quantity levels during HCoV infection like SARS and 2019-CoV. Conversely, SARS-CoV frequently targets for cytotoxic T lymphocytes [26, 27]. Lymphopenia is one of hematological abnormalities during SARS-CoV infection, and lymphocyte counts could predict the severity and clinical outcomes [28]. Previous study showed that lymphocytes and its subsets significantly decreased in SARS patients, while those with severe clinical illness or those who died had more remarkable CD4+ and CD8+ lymphopenia [28]. Also, MERS-CoV could efficiently infected T lymphocytes from the peripheral blood and from human lymphoid organs and induced apoptosis in T lymphocytes [29]. Similar with SARS-CoV and MERS-CoV, 2019-nCoV infection also related with loss of lymphocytes, which was supported by Chinese guidelines [30, 31]. Thus, the MLR increased especially in patients with disease progression.

Homocysteine is a potent toxic agent that involved in oxidative stress and neurotoxicity promotion, endothelial dysfunction, and acceleration of the atherosclerotic process [32–34]. Emerging evidences revealed that hyperhomocysteinemia contributed to a spectrum of disease development, including cardiovascular disease, diabetes, chronic kidney disease and fatty liver disease [35–37]. Previous reports uncovered that homocysteine concentrations were greater in many virus infections including human immunodeficiency virus, hepatitis virus and human papilloma virus [38–40]. However, the roles of homocysteine in coronavirus infection have not been well illustrated. Based on our results, homocysteine concentrated in imaging progression patients and showed predictive value for imaging progression.

Our results also demonstrated that COVID-19 patients with period from onset to admission ≥ 4 days had lower risk to develop imaging progression on chest CT at first week after admission. On the one hand, patients with period over 4 days might have mild clinical symptoms, which in line with mild or slow progression of this disease. On the other hand, the period from onset to admission should be counted in the natural process of 2019-nCoV infection.

This study has some limitations. First, only mild type of COVID-19 patients was included, and severe type and life-threating types were excluded in this analysis. Second, MLR and age did not have powerful prognostic values for imaging progression on chest CT in our study. Therefore, we suggest that they be used in combination in clinical practice. Third, the follow-up period was short-term, more solid outcomes should be considered in future. And, subgroup analysis of category manifestation of imaging progression on chest CT should also be considered. Even though, age, MLR model, homocysteine and period from onset to admission might be useful for evaluating disease progression in COVID-19 patients.

## MATERIALS AND METHODS

### Ethic statement

All participants provided written informed consent during their admission. The study protocol and informed consent documents were reviewed and approved by the Ethics Committee of Shanghai Public Health Clinical Center, Fudan University.

### Patients

In accordance to the 4^th^ edition of “Diagnosis and management program of novel coronavirus-infected pneumonia” released by National Health Commission of The People’s Republic of China [30], 273 diagnosed COVID-19 patients with mild category in Shanghai Public Health Clinical Center were included in this analysis. 2019 novel coronavirus (2019-nCoV) nucleic acid of sputum samples from all participants were positive detected by real-time polymerase chain reaction. The influenza A and B antigens of all participants were negative. All participants had no other lymphatic system disorders or malignant hematologic diseases, ensuring that the whole blood parameters were representative of normal baseline values. Patients with renal and/or hepatic failure, acute coronary syndromes, valvular heart diseases, autoimmune thyroid diseases, or systematic inflammatory diseases were excluded from our study.

### Study design

This was a prospective single-center cohort study. The baseline characteristics, including demographics, treatment strategies, routine blood tests, liver-kidney function parameters, coagulation function tests, cardiac markers and chest CT imaging, were all collected at the first admission day. Chest CT imaging were also performed at the (7 ± 2) day during their admission. All the tests and examines were conducted in the Department of Medical Laboratory and the Department of Radiology in Shanghai Public Health Clinical Center, Fudan University.

### Definition

The primary outcome was defined as imaging progression on chest CT at first week. Any one of the following criteria was considered as imaging progression on chest CT: 1) Increased ground-glass lesions in the underlying involvements; 2) Newly occurred lesions beyond underlying involvements. The chest CT imaging performance was diagnosed by two radiologists independently and inconsistency was discussed and determined by the director of Department of Radiology who acted as an arbiter.

Six inflammatory models, including ALRI, APRI, MLR, NLR, PLR and SII were included in this analysis. The definitions of these models are as follows: ALRI = AST / L; APRI = AST / P; MLR = M / L; NLR = N / L; PLR = P / L; and SII = P × N / L, where M, L, N and P are the peripheral monocyte, lymphocyte, neutrophil and platelet counts, respectively.

### Statistical analysis

Differences of variables between the individual groups were analyzed using student t test and Chi-square test based on variables types. Parameters associated with the outcome were assessed by univariate and multivariate logistic regression. Only variables significantly associated with the outcome at univariate analysis (two-sided p < 0.05) included in the multivariate model. Results were reported as risk ratios (RR) with 95% confidence intervals (CI). OptimalCutpoints package [41] in R program was used to perform ROC analysis to evaluate predictive values of potential factors for the outcome. Stata software version 16.0 (Stata Corp LLC, Texas, USA) was used for other statistics. A two-tailed p < 0.05 were considered significant for all tests.
